# Structure and Electrical Properties of Microwave Sintered BTS-BCT-*x*BF Lead-Free Piezoelectric Ceramics

**DOI:** 10.3390/ma15051789

**Published:** 2022-02-27

**Authors:** Tao Wang, Jian Ma, Bo Wu, Fenghua Wang, Shiyu Wang, Min Chen, Wenjuan Wu

**Affiliations:** 1 Sichuan Province Key Laboratory of Information Materials and Devices Application, Chengdu University of Information Technology, Chengdu 610225, China; handsomewang2020@163.com (T.W.); fhwimi@163.com (F.W.); wsy2019ktg@163.com (S.W.); chenmin@cuit.edu.cn (M.C.); 2 Sichuan Province Key Laboratory of Information Materials, Southwest Minzu University, Chengdu 610041, China; majian33@hotmail.com

**Keywords:** lead-free, BT-based ceramics, microwave sintered, structure, electrical properties

## Abstract

Barium titanate (BT)-based ceramics are one of the promising piezoelectric materials for environment-friendly electro-mechanical transformation. However, high performance materials are often sintered at high temperatures, resulting in volatile components and increased energy consumption. Here, 0.82Ba(Ti_0.89_Sn_0.11_)O_3_-(0.18-*x*)(Ba_0.7_Ca_0.3_)TiO_3_-*x*BiFeO_3_ (BTS-BCT-*x*BF) piezoelectric ceramics were prepared by microwave sintering (MWS) method, and the structure and properties were emphatically studied, aiming to reveal the regulatory mechanism of MWS on the structure and properties. Compared with conventional solid sintering (CS), the phase structure presents a similar evolution in MWS ceramics as a function of BF, while the more refined grain size and the denser structure are observed in MWS ceramics. The electrical properties (e.g., *d*_33_, *ε*_r_, tan *δ*, etc.) of MWS ceramics are superior to the CS ceramics owing to the refined grain size and denser microstructure. It is worth noting that the energy storage performance (e.g., energy storage density, energy storage efficiency) significantly outperformed expectations due to the slender hysteresis loop resulting from the smaller grain and high cubic phase. Therefore, the MWS sintering mechanism can further drive practical application of BT-based ceramics.

## 1. Introduction

As the representative of piezoelectric material, barium titanate (BT)-based ceramics are widely used in electronic components due to their excellent electric properties, such as micro-capacitors, ferroelectric memory, etc. [1,2]. However, the disadvantages, including high sintering temperature, poor temperature stability, and low breakdown field strength, seriously hinder the application in complex environment. In order to further seize the market, many means are used to overcome the shortages of BT-based materials, especially the various ways of preparation [3,4,5]. 

It is reported that the preparation of piezoelectric ceramics has many options in terms of sintering processes, such as spark plasma sintering (SPS) [6], atmosphere sintering [7], hot pressed sintering [8], microwave sintering (MWS) [9,10], etc. Among those options for sintering, MWS possesses the unique advantage of rapid sintering for materials at low temperature during the preparation process, which have been widely used to synthesize new materials [11,12]. It can also enhance the density of materials significantly, and improve the electrical properties. Takahashi H et al. reported a densified BT-based material system with high *d*_33_ prepared by MWS [13]. Bafandeh et al. prepared KNN-based ceramics by conventionally sintering and microwave-sintering, and systematically compared the microstructure, ferroelectric, and piezoelectric properties of those samples, showing that MWS can inhibit grain growth and enhance densification in this ceramic, which results in improvements in the electric properties (e.g., piezoelectric properties, ferroelectric properties, strain behavior, etc.) [14]. The effects of different sintering methods on the phase boundary and microstructure of BT-based ceramics were studied by Gao et al. [15]. Compared with conventional solid sintering (CS), the phase structure of microwave sintering ceramics remains almost stable, and the samples present a smaller grain size and uniform grain distribution. Another advantage of MWS is that it can significantly reduce temperature and time during the sintering process. However, the effects of MWS on the structure and properties have not been investigated sufficiently in BT-based ceramics with high performance. Revealing the regulatory mechanism of MWS on the structure and properties of BT-based ceramics is an urgent aim, which can further enrich strategies for tuning their performance.

Recently, a series of BT-based ceramics with excellent performance have been obtained by constructing multiphase boundaries though chemical modification (e.g., Ca, Sr, Zr, Sn, Hf, etc.). For example, Wang et al. [16] reported (Ba_0.85_Ca_0.15_)(Ti_0.9_Zr_0.1_)O_3_ (BCTZ) ceramics with large *d*_33_ (~650 pC/N), and Zhu et al. observed a high *d*_33_ in (Ba, Ca)TiO_3_–0.45Ba(Sn,Ti)O_3_ ceramics. In particular, Zhao et al. reported high piezoelectric properties (*d*_33_ = 700 ± 30 pC/N) in 0.82Ba(Ti_0.89_Sn_0.11_)O_3_-0.18(Ba_0.7_Ca_0.3_)TiO_3_ materials, which are comparable to that of lead-based and lead-free based piezoelectric materials. However, the ceramics with optimal piezoelectric properties were always sintered at a relatively high temperature, which could lead to the compositional volatile and increased the energy consumption during the sintering process. Therefore, it is expected that a lower temperature sintering could be used for fabrication, which will cut down the processing costs especially in production.

To further reveal the mechanism of MWS on the structure and properties BT-based ceramics with high performance, the Ba(Ti_0.89_Sn_0.11_)O_3_-(Ba_0.7_Ca_0.3_)TiO_3_ material system was chosen to verify the efficiency of this strategy in BT-based ceramics. In addition, BiFeO_3_ (BF), a typical ferroelectric material with large *P*_r_ and high *T*_c_, was also introduced to this material system, not only aiming to reduce the sintering temperature but also further modify the structure and properties [17,18,19]. For better comparison the effects of MWS and CS on the structure and properties, two sets of 0.82Ba(Ti_0.89_Sn_0.11_)O_3_-(0.18-*x*)(Ba_0.7_Ca_0.3_)TiO_3_-*x*BiFeO_3_ (BTS-BCT-*x*BF) samples were prepared by MWS and CS, aiming to reveal the regulatory mechanism of MWS and CS on the structure and electrical properties of lead-free piezoelectric ceramics, which may promote the practical application of BT-based ceramics.

## 2. Experimental Procedure

The 0.82Ba(Ti_0.89_Sn_0.11_)O_3_-(0.18-*x*)(Ba_0.7_Ca_0.3_)TiO_3_-*x*BiFeO_3_(BTS-BCT-*x*BF, *x* = 0.002, 0.004, 0.006, 0.008, 0.010, 0.012) piezoelectric ceramics were prepared by microwave sintering and normal sintering. Raw materials, including BaCO_3_ (99.0%), CaCO_3_ (99.0%), SnO_2_ (99.5%), TiO_2_ (98%), Bi_2_O_3_, and Fe_2_O_3_ (99%), were mixed with ZrO_2_ balls and the medium of ethanol for 12 h in planetary ball mill. After being dried in oven and calcined at 1200 °C for 2 h, these powders were mixed with polyvinyl alcohol (PVA, 7–8 wt%) and pressed into pellets of ~10 mm in diameter and ~1 mm in thickness. After the PVA binder burned out, the pellets were divided into two groups. One group was sintered at 1350 °C for 3 h by conventional sintering method, and the other group was sintered at 1290 °C for 2 h by microwave sintering method. Silver paste was used as electrodes for electrical measurements, and was fired at ~650 °C for 30 min. These samples were poled in a silicone oil bath under a dc field of ~3.0 kV/mm for 30 min.

X-ray diffractometer (XRD) with a *CuK*_α_ radiation (DX-2700, Haoyuan Instrument, Dandong, China) was used to confirm phase structure of the samples under ~40 kV, ~30 mA. Their microstructure and chemical compositions were measured by a field-emission scanning electron microscope (FE-SEM) (JSM-7500, JEOL, Showa, Japan). The dielectric constant (*ε*_r_) varying with temperature (−150–200 °C) was measured by using an LCR analyzer (HP 4980, Agilent, Palo Alto, USA) in connection with a temperature-controlled instrument. The polarization versus electric field (*P*-*E*) hysteresis loops and the strain-electric field (*S*–*E*) curves were measured at 1 Hz with a ferroelectric tester (Radiant Premier II, Radiant Technologies, Inc., Albuquerque, NM, USA). The *d*_33_ value was measured by a *d*_33_ m (ZJ-3A, Zhichuang Technology Development Co., Ltd., Beijing, China) for the poled samples. 

## 3. Results and Discussion

### 3.1. Phase Structure

**Figure 1:**
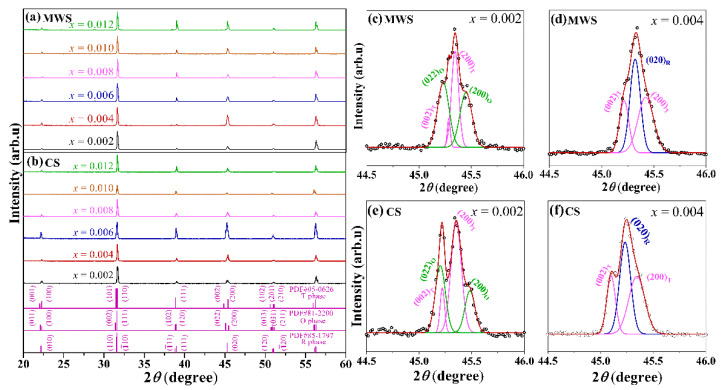
XRD patterns of BTS-BCT-*x*BF ceramics sintered with (**a**) MWS and (**b**) CS; enlarged XRD patterns with multi peak fitting of BTS-BCT-*x*BF ceramics with (**c**) *x* = 0.002, (**d**) *x* = 0.004 sintered by MWS; enlarged XRD patterns with multi-peak fitting of BTS-BCT-*x*BF ceramics with (**e**) *x* = 0.002, (**f**) *x* = 0.004 sintered by CS.

Figure 1a,b plots room temperature XRD patterns of BTS-BCT-*x*BF ceramics in the 2*θ* range of ~20–60° sintered with MWS and CS, respectively. Compared with the standard cards’ diffraction peaks of R (PDF # 85-1797), O (PDF # 81-2200), and T (PDF # 05-0626) in BaTiO_3_ materials, all ceramics show a pure perovskite structure without any other phase, indicating that BTS-BCT and BF formed multiphase ceramics within the study range of 0.002 ≤ *x* ≤0.012. In addition, the effect of BF content on the phase structure of BTS-BCT-*x*BF ceramics sintered with different sintering methods was analyzed by the enlarged XRD pattern with multi-peak fitting via Lorentz method (see Figure 1c,f) [20,21]. There are four separated peaks ((002)_T_, (200)_T_, (022)_O_ and (200)_O_) in the XRD pattern of the ceramics with *x* = 0.002, and three characteristics peaks ((020)_R_, (002)_T_ and (200)_T_) in the ceramics with *x* = 0.004, indicating that the sintering method did not change the phase structure of the ceramics. 

**Figure 2:**
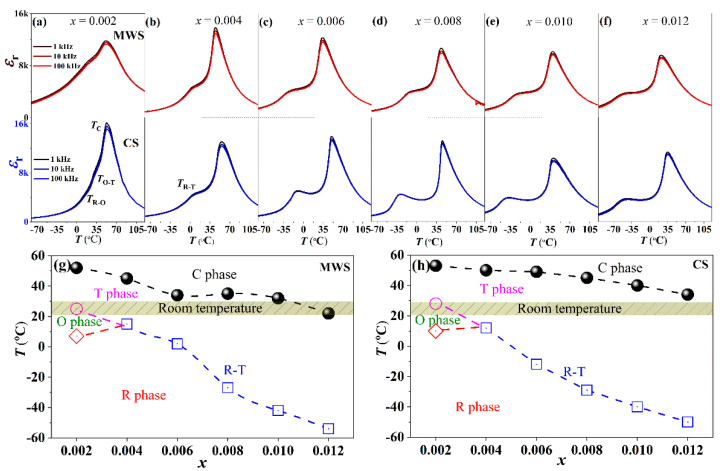
(**a**–**f**) *ε*_r-_*T* curves from −80 °C to 120 °C of BTS-BCT-*x*BF ceramics sintered with MWS and CS. (**g**,**h**) Phase diagrams of BTS-BCT-*x*BF ceramics sintered with MWS and CS, respectively.

Figure 2 shows the *ε*_r_-*T* curves (−80–120 °C) of BTS-BCT-*x*BF ceramics prepared by MWS and CS, measured at 1~100 kHz, which aimed to further verify the phase structure of BTS-BCT-*x*BF ceramics sintered with MWS and CS. Three dielectric peaks are observed in the curve of the ceramics with *x* = 0.002, corresponding to the rhombohedral-orthorhombic (R-O) phase transition temperature (*T*_R-O_) at lower temperature, the orthorhombic-tetragonal (O-T) phase transition temperature *(T*_O-T_) near room temperature, and the Curie temperature (*T*_c_) at high temperature, respectively. Only two dielectric peaks are observed in the curves of the ceramics with *x* > 0.002, which is related to the rhombohedral-tetragonal (R-T) phase transition temperature (*T*_R-T_) at lower temperature and Curie temperature (*T*_c_) at high temperature, respectively. One can see that the phase boundaries are very sensitive to BF content, as shown in Figure 2a–f. Compared with CS samples of *x* = 0.002, the *T*_O-T_ (MWS: ~25 °C, CS: ~28 °C) and *T*_R-O_ (MWS: ~6 °C, CS: ~10 °C) moves towards low temperature, and the *T*_c_ (MWS: ~52 °C, CS: ~53 °C) keeps almost stable in MWS ceramics, indicating that the O-T phase boundaries is obtained near room temperature. With increasing BF to 0.004, all ceramics show R-T phase boundaries because the O-T and R-O phases form a convergence zone near room temperature, and the *T*_R-T_ of MWS sample is higher than the CS ceramics. With further increasing BF, the *T*_R-T_ and *T*_c_ decrease simultaneously, and the T-C phase boundaries are observed near room temperature in those ceramics. In addition, *T*_c_ of MWS ceramics (52 °C→22 °C) changes faster than CS ceramics (53 °C→39 °C) in BTS-BCT-*x*BF ceramics. Figure 2g,h plots the phase diagram of MWS and CS BTS-BCT-*x*BF ceramics. The evolution of phase boundaries is highly matched with the XRD patterns, that is, O-T for 0.002 ≤ *x* < 0.004, R-T for 0.004 ≤ *x* < 0.006, and T-C for *x* ≥ 0.006. 

**Figure 3:**
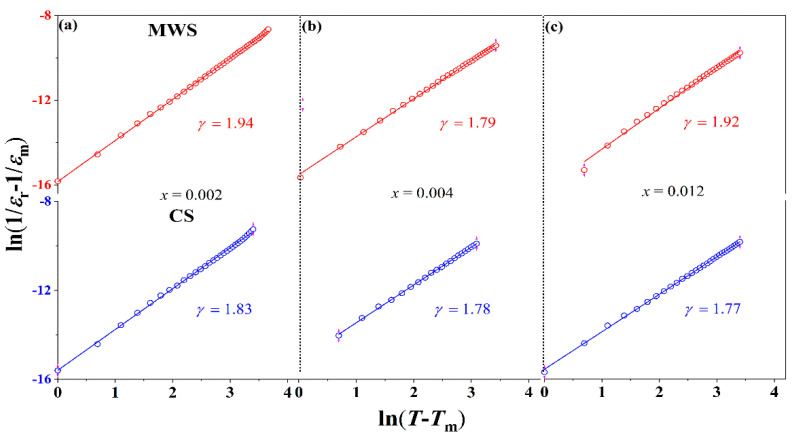
Plots of ln(1/*ε*_r_ − 1/*ε*_m_) versus ln(*T* − *T*_m_) of BST-BCT-*x*BF ceramics with MWS and CS, (**a**) *x* = 0.002, (**b**) *x* = 0.004, (**c**) *x* = 0.012.

Figure 3 plots the modified Curie-Weiss law (ln(1/*ε*_r_ − 1/*ε*_m_) − ln(*T* −*T*_m_)) of BST-BCT-*x*BF ceramic, which is originated from the following formula:

1/*ε*_r_ − 1/*ε*_m_ = (*T* − *T*_m_)^γ^/C, (1)

where *ε*_m_ is the maximum value of relative dielectric constant and γ is the degree of diffuseness. The higher γ value is observed in MWS ceramics, indicating that the MWS ceramics show a stronger relaxation degree than the CS ceramics. This can be ascribed to the smaller grain sizes in MWS ceramics, which will be discussed later.

### 3.2. Microstructure

**Figure 4:**
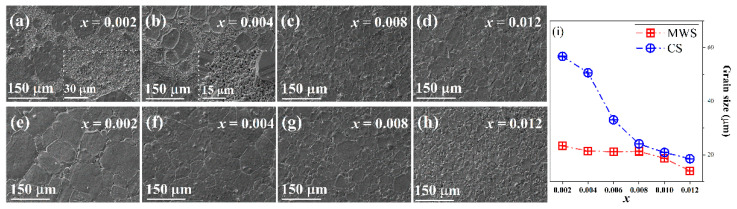
Surface morphology images of the BTS-BCT-*x*BF ceramics sintered by (**a**–**d**) MWS and (**e**–**h**) CS, (**i**) Grain sizes of MWS and CS BTS-BCT-*x*BF ceramics.

Figure 4a–d and Figure S1 (see the Supplementary Materials) show the SEM surface morphology of MWS ceramics. A phenomenon of bimodal distribution of large and small grains is observed in MWS ceramics with 0.002 ≤ *x* ≤ 0.004. The average grain size of large grains is about 43.65~47.96 μm, and the small grains of 1.0~1.8 μm surround the large grains, resulting in the improved density by filling the pores around the large grains, and the average grain size decreases from 29.14 μm to 23.60 μm. With further increasing BF in MWS ceramics, the average grain size decreases from 21.25 μm to 13.99 μm, and the bimodal distribution of grain disappears in those ceramics. Figure 4e–h shows the SEM of CS BTS-BCT-BF ceramics. It is found that the grain size tends to be smaller with the increase of BF content, that is, it decreases from 56.72 μm to 18.50 μm as a function of BF, suggesting that the excess BF can inhibit the grain growth. Compared with CS samples, the grain size is smaller in MWS ceramics (see Figure 4i), proving that the MWS method can also hinder the growth of grain in ceramics; such a phenomenon has been obtained elsewhere [22,23,24]. Significantly, the grain size of MWS BTS-BCT-*x*BF ceramics is close to CS with the increase of doping concentration starting from 0.008, while still a little smaller than the grain size of CS. This can be explained by the synthetic effect of MWS and high concentrations of BF. The effect of MWS has a major role in decreasing grain sizes at *x* < 0.008, and high concentrations of BF play an important role in decreasing grain sizes at *x* ≥ 0.008. In addition, the synthetic effect of MWS and high concentrations of BF on the grain sizes lead to a little smaller grain size in MWS than CS at *x* ≥ 0.008. In addition, the variation of relaxation degree can be ascribed to the evolution of grain size, as shown in Figure 3 and Figure 4. The decrease of grain size results in an increase of the internal stress induced by lattice distortion, which can change the forces between the short-range and long-range, thus the relaxation behavior of MWS ceramics is stronger than the CS. The density of the ceramics is measured through Archimedes drainage method, as shown in Table 1. One can find that the density first increases and then decreases in the samples as a function of BF, and the density of MWS ceramics is higher than CS ceramics, indicating that MWS plays a more effective role in enhancing density.

### 3.3. Dielectric and Ferroelectric Properties

**Figure 5:**
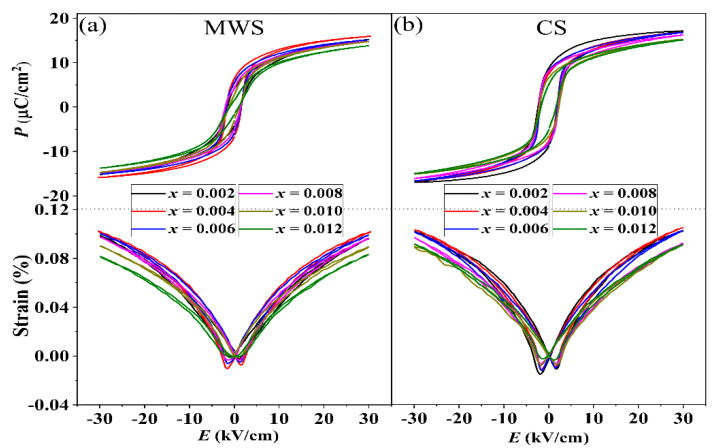
*P*-*E* and *S*-*E* loops of (**a**) MWS and (**b**) CS sintered BTS-BCT-*x*BF ceramics.

**Figure 6:**
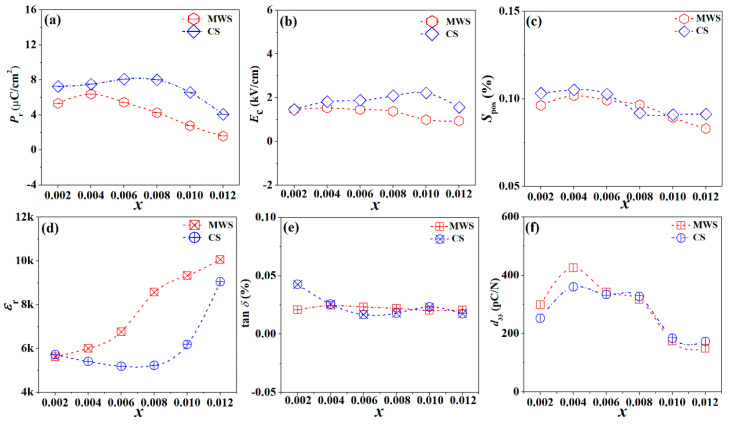
(**a**) *P*_r_, (**b**) *E*_c_, (**c**) *S*_pos_, (**d**) *ε*_r_, (**e**) tan *δ*, and (**f**) *d*_33_ of BTS-BCT-*x*BF ceramics sintered with MWS and CS.

Figure 5a,b shows the *P*-*E* and *S*-*E* loops for MWS and CS ceramics, measured at room temperature and 30 kV/cm. All samples present saturated *P*-*E* loops and standard *S*-*E* curves, indicating that all samples are normal ferroelectrics. Due to the high *P*_r_ for BF, the *P*_r_ increases with increasing the proper BF, and the ferroelectric properties would deteriorate in the ceramics with adding excessive BF, as shown in Figure 6a. The *P*_r_ of MWS samples is smaller than that of CS ceramics, which results from the grain refinement leading to a decrease in *P*_r_ due to the increased clamping effect of domain walls relative to grain boundaries [25,26]. Compared with the CS samples with *x* > 0.004, the *P*_r_ decreases sharply in MWS samples, which is related to the evolution of phase boundary. With increasing BF content, the cubic phase of MWS ceramics gradually dominates in the phase structure near room temperature, and then results in deteriorating of the ferroelectric properties. The *E*_c_ of two group samples keeps almost unchanged with increasing BF content, as shown in Figure 6b. The *S*_pos_ (the strain between the maximum strain and the strain under 0 kV/cm) of MWS and CS ceramics are shown in Figure 6c, which first increases and then decreases with increasing BF, achieving maximum *S*_pos_ value (MWS: ~0.102%, CS: ~0.105%) at the ceramics with *x* = 0.004, which can be ascribed to the low energy barrier in multiphase boundary. Moreover, the maximum strain in MWS ceramics is lower than CS due to the limitation of a large number of fine grain when *x* ≤ 0.004. Figure 6d plots the dielectric constant (*ε*_r_) of CS and MWS ceramics, measured at 10 kHz. The *ε*_r_ increases continuously in MWS ceramics, while first decreasing and then increasing in CS ceramics with increasing BF. The higher *ε*_r_ of MWS ceramics results from the finer grains because *ε*_r_ increases with decreasing grain size when the average grain size is greater than 1.2 µm [27,28]. Of course, the high density of MWS ceramics also contributes positively to this. The dielectric loss (tan *δ*) of two sets of samples is very close, and remains around 0.02~0.04, as shown in Figure 6e. This suggests that the tan *δ* is not sensitive to the sintering methods, and this phenomenon has been reported in other BT-based ceramics [29]. The *d*_33_ of MWS and CS ceramics presents a similar change trend, as shown in Figure 6f. That is, *d*_33_ first increases and then decreases with the increase of BF content, reaching the maximum values (MWS: ~425 pC/N, CS: ~360 pC/N) at *x* = 0.004. This can be attributed to the R-T phase boundaries near room temperature in the ceramic with *x* = 0.004. As is known, multiphase coexistence provides more possible polarization directions, resulting in easy polarization rotation, which can greatly facilitate the polarization switching [30]. Moreover, the bimodal grain sizes distribution can also enhance the *d*_33_ value in MWS ceramics [25].

### 3.4. Energy Storage Performance and Electrostriction

**Figure 7:**
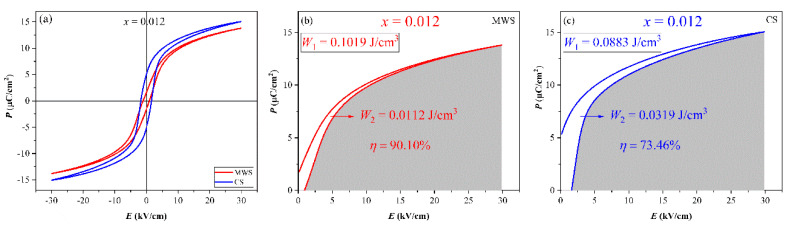
(**a**) P-E loops, energy storage performance of (**b**) MWS and (**c**) CS of BTS-BCT-*x*BF ceramics with *x* = 0.012.

**Figure 8:**
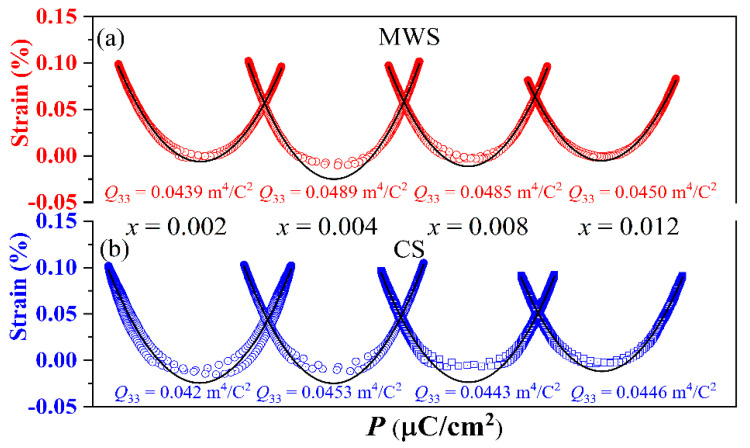
*S*-*P* loops of BST-BCT-*x*BF ceramics sintered with (**a**) MWS and (**b**) CS.

Figure 7a shows the *P*-*E* loops of the MWS and CS ceramics with *x* = 0.012. It is reported that the thinner *P*-*E* loop with high *P*_max_ and low *P*_r_ value is beneficial to energy storage due to the low energy loss in the materials. Compared with the *P*-*E* loops of CS ceramics, a thinner *P*-*E* loop is obtained in MWS ceramics, indicating that it has better energy storage performance than CS ceramics. To further make a quantitative analysis on the energy storage performance of MWS and CS ceramics, the calculated energy storage performance of MWS and CS ceramics with *x* = 0.012 are plotted in Figure 7b,c, measured at 30 kV/cm. As is known, the discharge storage density *W*_1_ can be obtained from the integration of the discharge curve, and the area between the charge Curve and the discharge curve is the energy loss *W*_2_, *η* = *W*_1_/(*W*_1_ + *W*_2_) is used to express energy storage efficiency [31,32,33]. Although the *P*_max_ of MWS ceramics is slightly lower than that of CS, the MWS ceramics have better energy storage density and energy storage efficiency (MWS: *W*_1_ = 0.1019 J/cm^3^, *η* = 90.10%; CS: *W*_1_ = 0.0883 J/cm^3^, *η* = 73.46%) due to the slender hysteresis loop resulting from the smaller grain and high cubic phase. Therefore, MWS may be beneficial to the enhanced energy storage performance. Polarization reveals that the strain is mainly contributed by electrostriction in the ceramics, and the *S*-*P* curves of some components deviate from the quadratic relationship caused by the irreversible domain switching [34,35,36,37,38,39]. The *Q*_33_ of the ceramics shows a similar trend, that is, *Q*_33_ increases first and then decreases, and reaches the maximum value at *x* = 0.004 (MWS: ~0.049 m^4^/C^2^, CS: ~0.045 m^4^/C^2^), as shown in Figure 8. Moreover, the MWS ceramics have higher *Q*_33_ than CS.

## 4. Conclusions

In this work, 0.82Ba(Ti_0.82_Sn_0.11_)O_3_-(0.18-*x*)(Ba_0.7_Ca_0.3_)TiO_3_-*x*BiFeO_3_ (BTS-BCT-*x*BF) piezoelectric ceramics were prepared by microwave sintering method and conventional sintering method. The phase structure of two group samples presents a similar evolution, and the more refined grain size was observed in MWS ceramics, resulting in the denser structure than CS ceramics. Owing to the refined grain size and denser microstructure in MWS ceramics, the electrical properties (e.g., *ε*_r_~8273, *d*_33_~425 pC/N, tan *δ*~0.022, Strain~0.102%, *Q*_33_~0.049 m^4^/C^2^) were better than the performance of CS ceramics (e.g., *ε*_r_~5400, *d*_33_~360 pC/N, tan *δ*~0.026, Strain~0.105%, *Q*_33_~0.045 m^4^/C^2^). In addition, the energy storage performance (e.g., energy storage density, energy storage efficiency) significantly outperformed expectations due to the slender hysteresis loop resulting from the smaller grain and high cubic phase, and the *Q*_33_ in MWS ceramics was also superior to the CS ceramics.

## Figures and Tables

**Table 1:** Density of BTS-BCT-*x*BF ceramics sintering by MWS and CS.

*x*	Sintering Method	Density (g/cm^3^)	Sintering Method	Density (g/cm^3^)
**0.002**	CS	5.8005	MW	5.8364
**0.004**		5.8285		5.9100
**0.006**		5.7959		5.8653
**0.008**		5.7644		5.8435
**0.010**		5.7721		5.8195
**0.012**		5.8059		5.8065

## Data Availability

The data that support the findings of this study are available within this article.

## Supplementary Materials

The following supporting information can be downloaded at: https://www.mdpi.com/article/10.3390/ma15051789/s1, Figure S1: Surface morphology images of the BTS-BCT-*x*BF ceramics sintered by (**a**,**b**) MWS and (**c**,**d**) CS.
