# Supplementary material for: Structure and Electrical Properties of Microwave Sintered BTS-BCT-xBF Lead-Free Piezoelectric Ceramics
Source: Materials (Basel). 2022 Feb 27;15(5):1789. doi: 10.3390/ma15051789 (PMC8911552; doi:10.3390/ma15051789)
Supplement: Supplementary file 1 [file materials-15-01789-s001.zip › materials-1552637-supplementary.pdf]

materials

MDPI

Supplementary Materials

Structure and Electrical Properties of Microwave Sintered BTS-BCT-*x*BF Lead-Free Piezoelectric Ceramics

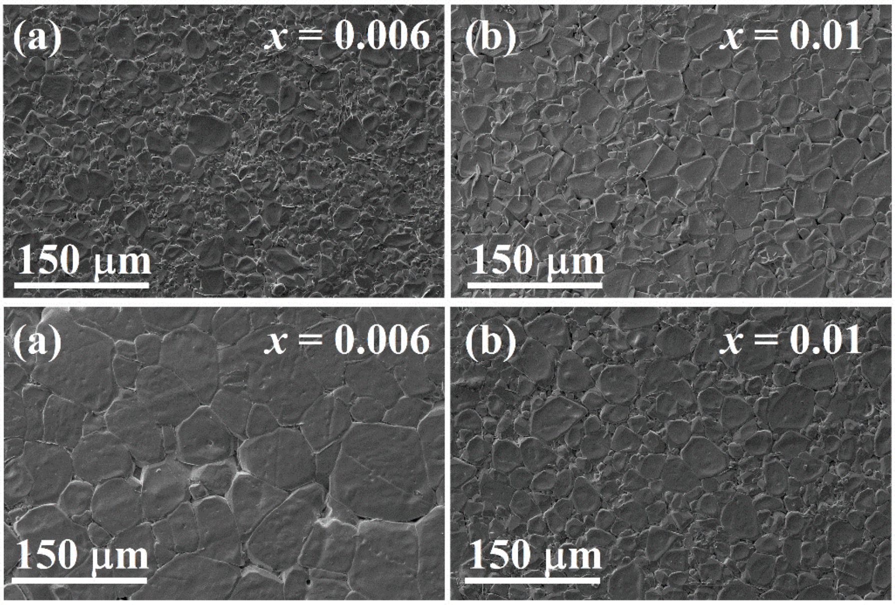

Figure S1. Surface morphology images of the BTS-BCT-*x*BF ceramics sintered by (**a**,**b**) MWS and (**c**,**d**) CS.

Citation: Wang, T.; Ma, J.; Wu, B.; Wang, F.; Wang, S.; Chen, M.; Wu, W. Structure and Electrical Properties of Microwave Sintered BTS-BCT-*x*BF Lead-Free Piezoelectric Ceramics. *Materials* **2022**, *15*, 1789. https://doi.org/10.3390/ma15051789

Academic Editor: Mattia Biesuz

Received: 28 December 2021
Accepted: 21 February 2022
Published: 27 February 2022

Publisher's Note: MDPI stays neutral with regard to jurisdictional claims in published maps and institutional affiliations.

Copyright: © 2022 by the authors. Submitted for possible open access publication under the terms and conditions of the Creative Commons Attribution (CC BY) license (https://creativecommons.org/licenses/by/4.0/).
